# Distribution of histone H4 modifications as revealed by a panel of specific monoclonal antibodies

**DOI:** 10.1007/s10577-015-9486-4

**Published:** 2015-09-05

**Authors:** Yoko Hayashi-Takanaka, Kazumitsu Maehara, Akihito Harada, Takashi Umehara, Shigeyuki Yokoyama, Chikashi Obuse, Yasuyuki Ohkawa, Naohito Nozaki, Hiroshi Kimura

**Affiliations:** Graduate School of Bioscience and Biotechnology, Tokyo Institute of Technology, 4259 Nagatsuta, Midori-ku, Yokohama, 226-8501 Japan; Graduate School of Frontier Biosciences, Osaka University, Suita, Osaka 565-0871 Japan; CREST, JST, Kawaguchi, Saitama 332-0012 Japan; Department of Advanced Medical Initiatives, Faculty of Medicine, Kyushu University, Fukuoka, 812-8582 Japan; RIKEN Center for Life Science Technologies, Yokohama, 230-0045 Japan; RIKEN Structural Biology Laboratory, Yokohama, 230-0045 Japan; School of Life Sciences, Hokkaido University, Sapporo, 001-0021 Japan; MAB Institute Inc, Sapporo, 001-0021 Japan

**Keywords:** Chromatin, Epigenetics, Histone modification, Monoclonal antibody

## Abstract

Post-translational histone modifications play a critical role in genome functions such as epigenetic gene regulation and genome maintenance. The tail of the histone H4 N-terminus contains several amino acids that can be acetylated and methylated. Some of these modifications are known to undergo drastic changes during the cell cycle. In this study, we generated a panel of mouse monoclonal antibodies against histone H4 modifications, including acetylation at K5, K8, K12, and K16, and different levels of methylation at K20. Their specificity was evaluated by ELISA and immunoblotting using synthetic peptide and recombinant proteins that harbor specific modifications or amino acid substitutions. Immunofluorescence confirmed the characteristic distributions of target modifications. An H4K5 acetylation (H4K5ac)-specific antibody CMA405 reacted with K5ac only when the neighboring K8 was unacetylated. This unique feature allowed us to detect newly assembled H4, which is diacetylated at K5 and K12, and distinguish it from hyperacetylated H4, where K5 and K8 are both acetylated. Chromatin immunoprecipiation combined with deep sequencing (ChIP-seq) revealed that acetylation of both H4K8 and H4K16 were enriched around transcription start sites. These extensively characterized and highly specific antibodies will be useful for future epigenetics and epigenome studies.

## Introduction

In eukaryotic nuclei, ∼150 bp of DNA is wrapped around the histone octamer, which consists of two copies of four core histones (i.e., H2A, H2B, H3, and H4) to form a nucleosome, the fundamental unit of chromatin. Post-translational modifications on these histones play a critical role in genome function, including the regulation of transcription and the maintenance of genome integrity (Bannister and Kouzarides 2011; Greer and Shi 2012; The ENCODE Project Consortium 2012; Jørgensen et al. 2013). Since H3 and H4 are more stably integrated into nucleosomes compared to H2A and H2B (Kimura and Cook 2001), the modifications on H3 and H4 can act as long-term memory of epigenetic regulation. Among various H3 modifications, trimethylation on H3 lysine 9 and 27 (H3K9me3 and H3K27me3) that is associated with silenced genes can be inherited over cell generations (Martin and Zhang 2005; Greer and Shi 2012; Kimura 2013). Other modifications, such as trimethylation on H3 lysine 4 (H3K4me3) and acetylation are associated with transcriptional activation (Heintzman et al. 2007; Shilatifard 2008; Stasevich et al. 2014). Modifications on histone H4 are also known to be involved in gene regulation and genome maintenance (Shahbazian and Grunstein 2007). Lysine residues in H4 N-terminal tail (i.e., H4K5, H4K8, H4K12, and H4K16) are major acetylation sites (Turner et al. 1989; Johnson et al. 1998; Lang et al. 2013; Taylor et al. 2013; Zheng et al. 2013). These acetylations are predominantly associated with euchromatin, contributing to chromatin decondensation and transcriptional regulation (Turner 1991; Dion et al. 2005; Wang et al. 2008; Bannister and Kouzarides 2011). H4K16ac is also known to be associated with DNA damage repair and cell senescence (Dang et al. 2009; Li et al. 2010; Sharma et al. 2010; Krishnan et al. 2011). In addition, H4K5ac and H4K12ac are associated with newly assembled chromatin since H4 in predeposition complexes is diacetylated at K5 and K12 by a histone acetyltransferase (HAT) (Sobel et al. 1995; Chang et al. 1997). Although the diacetylation of H4 is not a prerequisite for histone assembly (Ma et al. 1998), these modifications may stimulate nuclear import (Ejlassi-Lassallette et al. 2011) and contribute to the recovery from replication block-mediated DNA damage (Barman et al. 2006). After being assembled into chromatin, H4 becomes deacetylated in heterochromatin (Taddei et al. 1999). In contrast to these four lysines that are acetylated, H4 lysine 20 (H4K20) is subject to methylation (Jørgensen et al. 2013). Monomethylation of H4K20 (H4K20me1) may play multiple roles in genome regulation, including transcriptional control, DNA replication licensing, DNA damage response, and chromosome segregation (Wu and Rice 2011; Beck et al. 2012; Kapoor-Vazirani and Vertino 2014). Dimethylation of H4K20 (H4K20me2) is one of the most abundant modifications in mouse fibroblasts and HeLa cells (Pesavento et al. 2008; Schotta et al. 2008), and is involved in DNA damage repair signaling (Greeson et al. 2008).

Trimethylation on H4 lysine 20 (H4K20me3) is also involved in heterochromatin formation, correlated with H3K9me3 (Schotta et al. 2004; Sims et al. 2006). The levels of some histone H4 modifications, like H4K16ac and H4K20me3, are reported to be altered in cancer cells, suggesting these marks can possibly be diagnostic markers (Fraga et al. 2005; Ellis et al. 2009; Rodriguez-Paredes and Esteller 2011; Yokoyama et al. 2014).

To analyze histone modifications, specific antibodies have been valuable tools with a variety of applications, such as chromatin immunoprecipitation, immunoblotting detection, and immunofluorescence (Turner et al. 1992; Peters et al. 2003; Kimura et al. 2008). The quality and reproducibility of these immunochemical analyses rely on antibody specificity and affinity, but commercially available antibodies have not been validated extensively (Clayton et al. 2006; Kimura et al. 2008; Rothbart et al. 2012; Kimura 2013). For example, 20–25 % of histone modification-specific antibodies failed to pass the validation by the ENCODE project (Egelhofer et al. 2011). To facilitate chromatin and epigenetics studies, we have developed histone H3 modification-specific antibodies (Kimura et al 2008; Hayashi-Takanaka et al. 2009, 2011, 2014; Chandra et al. 2012). We here report the generation and validation of a panel of monoclonal antibodies (mAbs) directed against histone H4. These highly reliable and fully characterized mAbs will be useful for future work.

## Materials and methods

### Ethics statement

All institutional and national guidelines for the care and use of laboratory animals were followed. All animal care and experimental procedures in this study were approved by the Hokkaido University Animal Experiment Committee (approval number: 11-0109) and carried out according to guidelines for animal experimentation at Hokkaido University, where Mab Institute Inc is located. Animals were housed in a specific pathogen-free facility at Hokkaido University. Humane euthanasia of mice was performed by cervical dislocation by individuals with a demonstrated high degree of technical proficiency.

### Generation, selection, purification, and dye conjugation of monoclonal antibodies

**Fig. 1:**
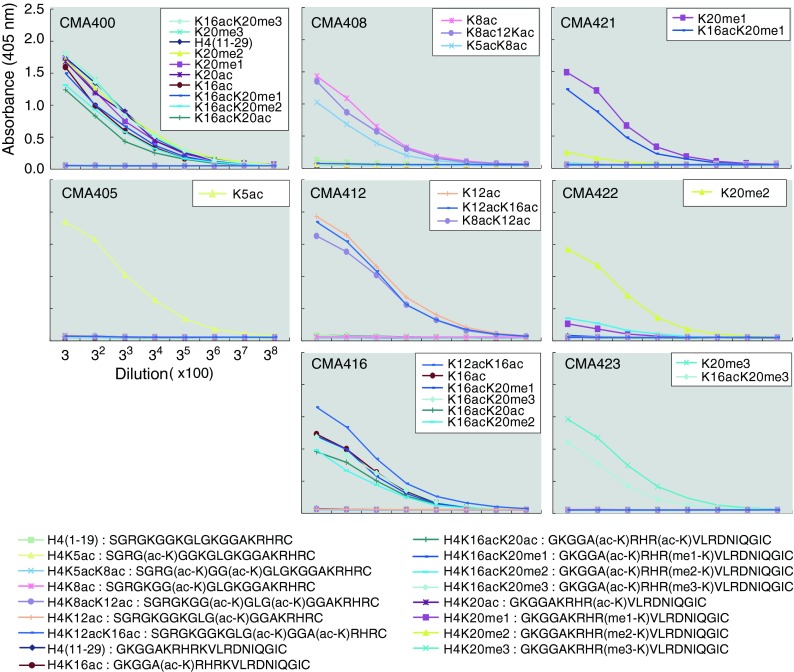
Specificity of mAbs evaluated by ELISA. Microtiter plates coated with the indicated peptides conjugated with bovine serum albumin were incubated with threefold dilutions of each antibody, starting from 1:100 dilution of a hybridoma culture supernatant. After incubation with peroxidase-conjugated secondary antibody and washing, the colorimetric signal of tetramethylbenzidine was detected by measuring the absorbance at 405 nm using a plate reader. The peptides reacted with the individual mAb are indicated on the panel

Synthetic peptides (Sigma-Genosys; Fig. 1) were coupled to keyhole limpet hemocyanin and used to immunize mice (Kimura et al. 1994); after generating hybridomas, clones were screened by ELISA using plates coated with the modified or unmodified peptide conjugated with bovine serum albumin. After recloning, supernatants from clones reacting with the specifically modified peptide were used to probe blots prepared using HeLa total proteins, and the ones giving a single band at the size of histone H4 were selected for immunofluorescence examination to see if they exhibited nuclear staining. Clones that passed through these successive screens were further analyzed by ELISA against peptides listed in Fig. 1, and the ones showing the highest specificity to the authentic peptides were selected. The isotype of each mAb was determined using a kit (Serotec; MMT1; Table 1).

**Table 1:** Summary of monoclonal antibodies

Name	Residue	Modification	Subclass	Allowance	Occlusion
CMA400 (200a 9C5)	H4 (21–29)	Unmodified	IgG2b-κ	K20-methyl	
CMA405 (200Nb 4A7)	K5	Acetyl	IgG1-κ		K8-acetyl
CMA408 (200Nd 72A9)	K8	Acetyl	IgG1-κ	K5-acetyl K12-acetyl	
CMA412 (200Nf 50B3)	K12	Acetyl	IgG1-κ	K8-acetyl K16-acetyl	
CMA416 (200f 1B2)	K16	Acetyl	IgG1-κ	K12-acetyl K20- acetyl K20- methyl	
CMA421 (200b 15F11)	K20	Monomethyl	IgG1-κ	K16-acetyl	
CMA422 (200c 2E2)	K20	Dimethyl	IgG1-κ		K16-acetyl
CMA423 (200d 27F10)	K20	Trimethyl	IgG1-κ	K16-acetyl	

Hybridoma cells were routinely grown in GIT medium (Wako). For antibody purification, cells were grown in CD Hybridoma medium (Life Technologies) and the supernatant (250 ml) was filtrated through a 0.45-μm pore filter, as previously described (Hayashi-Takanaka et al. 2011). For IgG1 subclass mAbs, NaCl was added at a final concentration of 4 M before applying to a HiTrap Protein A HP Sepharose column (1 ml; GE Healthcare), which were pre-equilibrated with Protein A IgG1 binding buffer (Thermo Fisher Scientific). After washing the column with Protein A IgG1 binding buffer, IgG1 was eluted using Mouse IgG1 Mild Elution Buffer (Thermo Fisher Scientific). For IgG2b mAbs, the filtrated supernatant was directly loaded to a HiTrap Protein A HP Sepharose column (1 ml; GE Healthcare) pre-equilibrated with phosphate-buffered saline (PBS). After washing with PBS, IgG was eluted using IgG Elution buffer (Thermo Fisher Scientific) and the buffer pH was immediately neutralized using 1.5 M Tris–HCl (pH 8.8). IgG was concentrated up to ∼2 mg/ml in PBS using an Amicon Ultra filter (50 k-cut off; Millipore).

Purified IgG was conjugated with a fluorescent dye (Hayashi-Takanaka et al. 2014). Dried Alexa Fluor 488 5-SDP ester (1 mg; Invitrogen), Cy3 *N*-hydroxysuccinimide ester (for labeling 1 mg protein; GE Healthcare), and Cy5 *N*-hydroxysuccinimide ester (for labeling 1 mg protein; GE Healthcare) were dissolved into 100 μl dimethyl sulfoxide (DMSO; Wako) and stored at −20 °C. IgG (100 μg) was diluted into 100 mM NaHCO3 (pH 8.3) in 100 μl. After addition of a dye solution (1, 1.5, and 4 μl for Alexa488, Cy3, and Cy5, respectively), the mixture was incubated for 1 h at room temperature with gentle rotation. The sample was passed through a PD-mini G-25 desalting column (GE Healthcare), which was pre-equilibrated with PBS, to remove unconjugated dyes, and dye-conjugated IgG was concentrated up to ∼1 mg/ml using an Ultrafree 0.5 filter (10 k-cut off; Millipore). The IgG concentration and dye:protein ratio were calculated from the absorbance at 280 and 494, 552, or 650 nm, using the extinction coefficient of IgG and correction factor at 280 nm provided by the manufacturers. Fluorescent dye-labeled IgG samples that yielded dye:protein ratio ∼3–9 were used for live imaging.

### Cells

hTERT-RPE1 (purchased from Clontech; September 22, 1999; the pseudodiploid karyotype occasionally checked with most recently in February 2015) and HeLa cells (obtained from Peter Cook; ref. Pombo et al. 1999) were grown in Dulbecco’s Modified Eagle’s medium (DMEM; Nacalai Tesque) supplemented with l-glutamine/penicillin/streptomycin (2 mM l-glutamine, 100 U/ml penicillin, 0.1 mg/ml streptomycin; Sigma-Aldrich) and 10 % fetal calf serum (FCS), as previously described (Hayashi-Takanaka et al. 2009).

For ChIP experiments, mouse embryonic stem cells (ZHBTc4 cell line) were obtained from RIKEN BRC on January 11, 2011 (Niwa et al. 2000). Cells were maintained on culture dishes coated with 0.1 % type-A gelatin (Sigma-Aldrich) in Glasgow Minimum Essential Medium (Sigma-Aldrich) supplemented with 10 % fetal bovine serum (Equitech-Bio, Kerrville, TX), 1× nonessential amino acids (Nacalai Tesque), 1 mM sodium pyruvate (Nacalai Tesque), 0.1 mM 2-mercaptoethanol (Nacalai Tesque), and 2000 U/ml Murine LIF.

### Immunoblotting

Acetylated H4 tagged with NHis was produced by an in vitro translation system and purified as described (Mukai et al. 2011). Methylation mimic histones were purchased (Active Motif). HeLa histones were purified using the Histone Purification mini kit (Active Motif). H4 proteins (100 and 10 ng for Coomassiee-staining and immunoblotting, respectively) were separated in 15 % SDS-polyacrylamide gels (Wako), and either stained with Coomassiee Brilliant Blue (Wako) or transferred onto PVDF membranes (Pall) for immunoblotting using a semi-dry system (ATTO; 192 mM glycine, 100 mM Tris, and 5 % methanol; 70 min). PVDF membranes were washed with TBST (20 mM Tris–HCl (pH 8.0), 150 mM NaCl, and 0.05 % Tween 20), blocked with Blocking One-P (Nacalai Tesque) for 20 min, and incubated in mAbs (0.1–0.2 μg/ml) in TBST containing 10 % Blocking One-P for 1 h. After washing with TBST four times over 30 min, membranes were incubated in peroxidase-conjugated anti-mouse IgG (GE Healthcare; 1:1,000 in TBST) for 1 h, washed with TBST four times over 30 min, and soaked in chemiluminescent reagent (Perkin-Elmer). Chemiluminescent signals were captured using a LAS-3000 (Fuji film).

Expression vectors for lysine-to-alanine substitution mutants were constructed based on pBOS-H4-N-GFP (Kimura and Cook 2001) using a Quickchange kit (Stratagene) with specific primers containing single mutations. The resulting constructs were verified by nucleotide sequencing. HeLa cells grown on a 60-mm dish were transfected with plasmid DNA (2 μg) using Lipofectamine 2000 (Life Technologies; 10 μl) and further grown for a day. Cells were lysed using 2× SDS-gel loading buffer (0.5 ml) and boiling for 5 min, and 5 μl of lysate was separated in 10–20 % SDS-polyacrylamide gels (Wako) for immunoblotting.

### Immunofluorescence

Cells were fixed, permeabilized, and blocked as described previously (Kimura et al. 2006; Hayashi-Takanaka et al. 2009). To label replication foci, cells were incubated in 10 μM EdU for 7.5 min before fixation, and the signal was detected with Alexa Fluor 647 azide using a Click-iT EdU Imaging Kit (Life Technologies).

Cells were then incubated in labeled mAbs (0.2–1 μg/ml) with Hoechst 33342 (100 ng/ml in PBS) in PBS containing 10 % Blocking One-P (Nacalai Tesque) for 2 h at room temperature. After washing with PBS, coverslips were mounted in Prolong Gold (Life Technologies). Fluorescence images were collected using a confocal microscope (FV-1000; Olympus) operated by the built-in software (ver 2.1-4.1) with a PlanSApo 60× (NA = 1.35) or a PlanApoN SC 60× (NA = 1.4) objective lens (512 × 512 pixels; 12.5 μs/pixel; 4 line Kalman; 12-bit; pinhole 110 μm). Hoechst 33342, Alexa488, Cy3, and Cy5/Alexa647 signals were acquired by sequential scanning using laser lines at 405, 488, 543, and 633 nm, combined with emission dichroic mirror/barrier filter SDM490/BA430-470, SDM560/BA505-525, SDM640/BA560-620, and none/BA650IF, respectively, with a main dichroic mirror DM405/488/543/633.

### ChIP

Cells (ZHBTc4) were cross-linked in 0.5 % formaldehyde and suspended in 250 μl ChIP buffer (5 mM PIPES (pH 8.0), 200 mM KCl, 1 mM CaCl2, 1.5 mM MgCl2, 5 % sucrose, 0.5 % NP-40, and protease inhibitor cocktail; Nacalai Tesque). Samples were incubated for 15 min on ice, sonicated for 5 s three times, and digested with micrococcal nuclease (1 μl; New England Biolabs) and Ribonuclease A (1 μl; Nacalai Tesque) at 37 °C for 40 min. The digested samples were centrifuged at 15,000×*g* for 10 min. Supernatant containing 4–8 μg DNA was incubated with a mouse monoclonal antibody against H4K5ac, H4K8ac, H4K12ac, or H4K16ac, pre-bound to magnetic beads at 4 °C overnight with rotation. The immune complexes were washed twice with ChIP buffer and twice with TE buffer. After, cross-links were reversed by incubating in TE containing 1 % SDS at 65 °C overnight with proteinase K, and DNA was purified using a QIAquick PCR purification kit (Qiagen).

### ChIP-seq data analysis

Sequenced reads of chromatin immunoprecipiation combined with deep sequencing (ChIP-seq) data were mapped onto the mouse genome (mm9) with Bowtie (version 0.12.8) using the parameter “-n 3 -m 2”. Estimation of normalized ChIP-seq signal intensities were calculated as follows. First, we counted mapped reads at 10,000 bp (Fig. 4a, b), 200 bp (Fig. 4c), or 100,000 bp (Fig. 4d) intervals (bins) on the genome. The counts were normalized as RPKM (reads per kilobase per million mapped reads) (Mortazavi et al. 2008). Net ChIP-seq signal intensities were obtained by subtracting the input RPKM in each bin (Accession number, DRA002438 for H4 acetylation data; and SRX185820 for H3K7ac data).

### mRNA-seq and data analysis

Sequencing was performed using a Genome Analyzer IIx (Illumina). PolyA+ RNA was enriched from 100 ng of total RNA by two successive rounds of oligo(dT) selection. The polyA+ RNA was fragmented and first-strand cDNA was synthesized by random hexamer priming. Following second-strand cDNA synthesis, dsDNA was repaired using T4 DNA polymerase, Klenow enzyme, and T4 polynucleotide kinase (PNK) (New England Biolabs), followed by treatment with Klenow exo- to add an A base to the 3′ end. After ligation of the adaptor using TaKaRa Ligation Mix (TaKaRa), the adaptor-ligated DNA was amplified using PCR primers for 12 cycles, and the amplified library was isolated using the E-gel Electrophoresis System (Life Technologies). The samples were purified using a QIAquick MinElute Kit (Qiagen). Sequenced reads were mapped onto the mouse genome (mm9) using Tophat (version 2.0.10). Gene expression levels (FPKM; Fragments per kilobase of exon per million mapped sequence reads) were estimated by Cufflinks (version 2.1.1) using the parameters “-u -b (on cuffdiff)”. We classified genes into 11 groups based on the expression levels: Silent (FPKM = 0) and 10 groups with different expression levels separated at 10 percentile intervals (q0–10 %, q10–20 %, … and q90–100 % of genes from the lowest to the highest FPKM).

For a close look at ChIP-seq results, IGV_2.0.34 was used, as in Fig.4a, b.

## Results and discussion

### Generation of mAbs directed against modified histone H4

We generated mAbs directed against unmodified and specifically modified forms of histone H4, including acetylation at K5, K8, K12, and K16, and three levels of methylation at K20 (Table 1). The specificity was evaluated by ELISA and immunoblotting using synthetic peptides and recombinant proteins, respectively.

### Pan-H4 antibody for immunoblotting

**Fig. 2:**
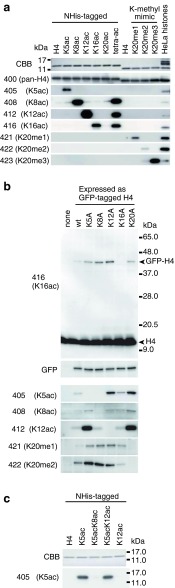
Specificity of mAbs evaluated by immunoblotting. **a** mAb reactivity to recombinant and HeLa histone H4. NHis-tagged H4, NHis-tagged acetylated H4 (monoacetylated or tetraacetylated at K5/K8/K12/K16 [tetra-ac]), H4, methyl-mimic H4, and HeLa histones were separated in 15 % SDS-polyacrylamide gels and stained with Coomassiee Brilliant Blue (*top panel*) or probed with the indicated mAbs (*lower panels*). The positions of size standards are indicated on the left of the top panel. **b** mAb reactivity to GFP-H4 mutants. GFP-H4 (wild-type; wt) and lysine-to-alanine (KA) mutants were expressed in HeLa cells. Total protein was separated in 10–20 % SDS-polyacrylamide gel and probed with the indicated mAbs. The *top panel* with anti-H4K16ac mAb CMA416 shows a typical example. Both the endogenous H4 and GFP-tagged H4 and KA mutants except K16A are detected with CMA416. The positions of size standards are indicated on the right. The membrane was reprobed using GFP-specific antibody (*second panel*). Relevant parts are shown for other mAbs (*lower panels*). **c** CMA405 reactivity to recombinant histone H4. NHis-tagged H4 proteins were separated in 15 % SDS-polyacrylamide gels and stained with Coomassiee Brilliant Blue (*top panel*) or probed with CMA405 (*bottom panel*). The positions of size standards are indicated on the right

mAb CMA400 reacted with all peptides containing amino acid 11–29 of H4, regardless of modifications at K16 and K20, but not with those containing amino acid 1-19 (Fig. 1). This indicates that CMA400 recognizes the peptide sequence between amino acid 21 and 29. Consistently, immunoblotting showed that CMA400 reacts with all recombinant H4 proteins harboring acetylation at K5, K8, K12, K16, and K20, and methylation mimic modifications at K20 as well as H4 prepared from HeLa cells (Fig. 2a). Therefore, CMA400 appears to be useful as a pan-H4 mAb by immunoblotting. CMA400, however, failed to stain cell nuclei by immunofluorescence, possibly because the epitope region is masked due to the direct interaction between the H4 tail in a nucleosome and H2A in another nucleosome (Luger et al. 1997; Sinha and Shogren-Knaak 2010).

### Acetylation-specific antibodies

ELISA and immunoblotting also revealed that modification-specific mAbs reacted exclusively with the corresponding peptides and recombinant H4 harboring the target modifications (Figs. 1 and 2). For example, CMA416 reacted with all peptides containing K16ac, regardless of the neighboring modifications such as K12ac, K20ac, and K20me1-3 by ELISA (Fig. 1). The specific binding of CMA416 to H4K16ac was confirmed by immunoblotting using recombinant H4; it highlighted H4 proteins harboring K16 acetylation and K5/K8/K12/K16 tetra-acetylation (Fig. 2a). In addition to the endogenous H4, exogenously expressed GFP-tagged H4 and mutant proteins, except H4K16A, were detected by CMA416 (Fig. 2b), indicating that H4K16 is required for CMA416 binding. From these data, it is concluded that CMA416 specifically binds to H4K16ac. Similarly, CMA408 and CMA412 specifically reacted with H4K8ac and H4K12ac, respectively, regardless of the neighboring modifications (Figs. 1 and 2).

Interestingly, CMA405 was specific to H4K5ac combined with unmodified K8, distinguishable from diacetylated H4 at K5 and K8 (Figs. 1 and 2). In ELISA, CMA405 reacted with monoacetylated peptide at K5 but not with diacetylated peptide at K5 and K8 (Fig. 1). Immunoblotting also showed that CMA405 exclusively reacted with K5-acetylated H4, but not with tetraacetylated H4 at K5/K8/K12/K16 (Fig. 2a), or H4K8A and H4K5A mutants (Fig. 2b). These results indicate that both acetylated K5 and unacetylated K8 are required for CMA405 binding. In Fig. 2b, CMA405 reacted more with H4K12A and H4K20A mutants, suggesting that CMA405 reactivity depends on these residues. To examine whether K12 acetylation influences the antibody recognition, we used recombinant histone H4 that harbors both K5 and K12 acetylation. As shown in Fig. 2c, CMA405 reacted equally with K5-acetylated and K5/K12-diacetylated H4, but not with K5/K8-diacetylated and K12-acetylated H4, indicating that CMA405 reactivity is not affected by K12 modification state. The differential reactivity to transiently expressed H4K12A mutant can be explained by the interplay between the modifications of different residues in living cells, as also observed in H4K12 acetylation (Fig. 2b; CMA412 blot) and in a previous study (Sasaki et al. 2009).

**Fig. 3:**
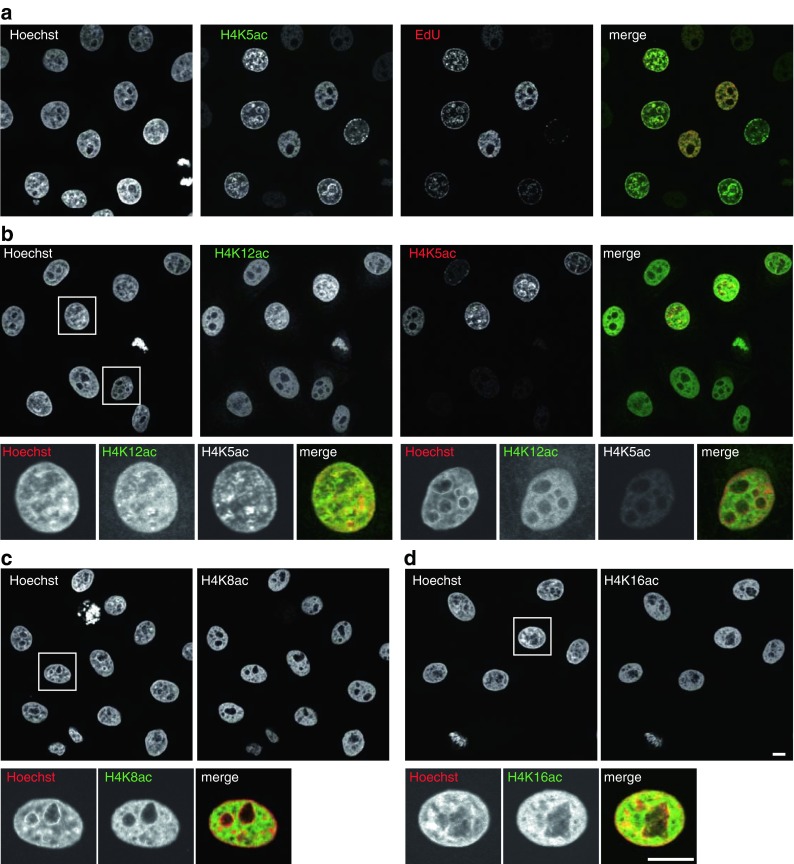
Localization of acetylation-specific mAbs. HeLa cells were fixed and immunolabeled with mAbs conjugated with Alexa Fluor 488 and Cy3. DNA was counterstained with Hoechst 33342. Shown are single confocal sections. **a** To label replication foci, cells were incubated with EdU for 7.5 min before fixation. EdU was detected with Alexa Fluor 647-azide before immunolabeling with H4K5-specific CMA405 conjugated with Alexa488. CMA405 overlaps with EdU (replication) foci in S phase cells. **b** H4K12ac-specific CMA412 (Alexa488) generally distributes in euchromatin and also enriched in CMA405 (Cy3) foci. **c** H4K8ac-specific CMA408 (Alexa488) is enriched in euchromatin. **d** H4K16ac-specific CMA416 (Alexa488) is enriched in euchromatin. *Bars*, 10 μm

The unique characteristic of CMA405 appeared to selectively detect newly assembled H4, which is diacetylated at K5 and K12 (with unacetylated K8) over the hyperacetylated H4, which harbors acetylation on K5, K8, K12, and K16. Indeed, immnofluorescence revealed that only a fraction of cells were intensely stained with CMA405, and the intra-nuclear distribution resembled replication foci in S phase cells (Fig. 3a). To compare the distribution of CMA405 with replication foci, cells were pulse-labeled with ethynyl-deoxyuridine (EdU) for 7.5 min, fixed, and stained with Alexa Fluor 647-azide and CMA405. As shown in Fig 3a, CMA405 overlapped with Alexa647-labeled EdU, confirming that CMA405 detected newly assembled histone H4 that harbor K5 acetylation. CMA412 was also enriched in replication foci but still remained in euchromatin where the signal did not overlap with Hoechst (Fig. 3b). This is explained by the specificity of CMA412, which reacted with H4K12ac regardless of the neighboring K8 and K16 acetylation (Fig. 1). CMA412 could detect both newly assembled H4, which harbors acetylated K12 with unacetylated K8 and K16, and euchromatin-associated H4, which harbors acetylation at K8 and/or K16 together with K12. Immunofluorescence further revealed that CMA408 and CMA416 showed a euchromatin distribution, consistent with a role of H4K8 and H4K16 acetylation in chromatin decondensation and transcriptional activation (Fig. 3c, d).

### ChIP-seq with acetylation-specific antibodies

**Fig. 4:**
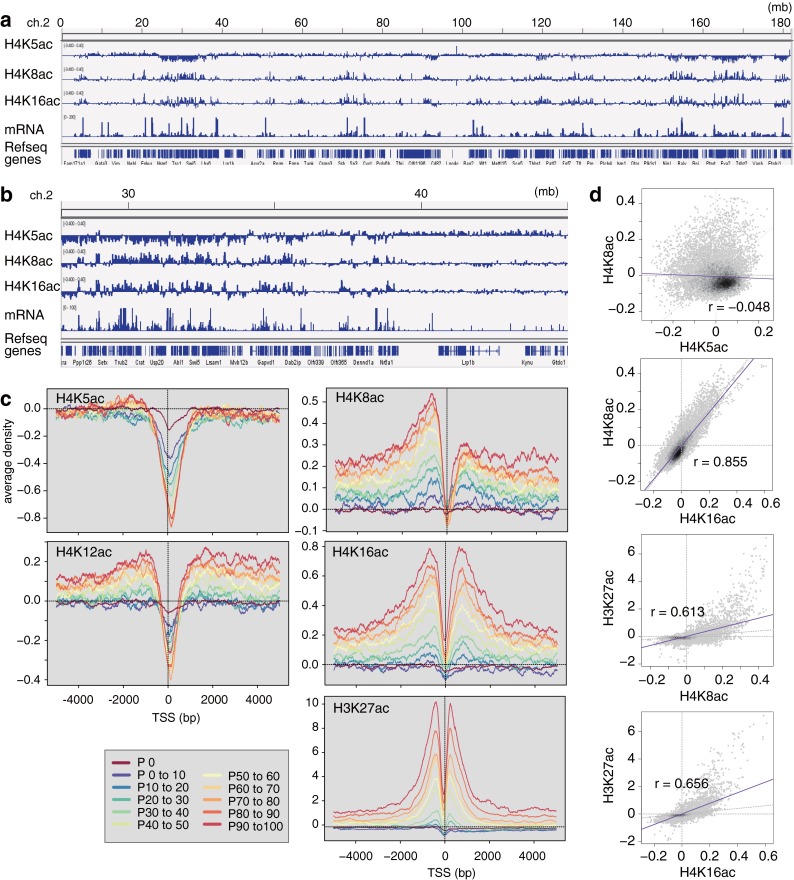
Localization of acetylated H4 in mouse ES cells by ChIP-seq. Genome-wide distribution of acetylated H4 was analyzed by ChIP-seq using CMA405, CMA408, CMA412, and CMA416. **a** Snapshot of the enrichment in whole chromosome 2. **b** Snapshot of the enrichment in chromosome 2 with a narrow view field. **c** Aggregation plots. Normalized peak counts of H4K5ac, H4K8ac, H4K12ac, H4K16ac, and H3K27ac signals surrounding the transcription start site (TSS) are indicated for 11 groups of genes which were divided based on their gene expression. **d** Correlation among histone H4K5, K8, K16, and H3K27ac modifications. H4K8ac, K16, and H3K27ac have positive correlation but not with H4K5ac

To reveal the localization of acetylation at different lysines in H4, we performed ChIP-seq analysis using mouse embryonic stem cells with the specific antibodies. In a genome-wide view, H4K5ac was not enriched in specific loci, but was rather depleted from transcriptionally active regions, complementary to H4K8ac and H4K16ac (Fig. 4a, b). Aggregation plots confirmed that H4K5ac with unacetylated K8 (detected by CMA405) was excluded from transcription start sites (TSSs) of actively transcribed genes (Fig. 4c). This depletion in highly expressed genes probably reflects the absence of nucleosomes at TSSs and the presence of hyperacetylated H4, in which K5 and K8 are both acetylated, around TSSs. H4K12ac (detected by CMA412) was distributed similarly to H4K5ac, but slightly enriched in upstream and downstream regions from TSSs in expressed genes. This is consistent with the property of the antibody, which binds to H4K12ac regardless of acetylation at K8 and K16, and detects both replication-associated and euchromatin-associated H4K12ac. H4K8ac and H4K16ac (detected by CMA408 and CMA416, respectively) were enriched around the TSS of highly expressed genes like H3K27ac, but their distributions were broader and slightly more distant from the TSS than H3K27ac. Correlation analysis also indicated the high similarity between H4K8ac and H4K16ac (Fig. 4d). H3K27ac and H4K8ac, or H4K16ac, were also correlated, but not as much as the correlation between H4K8ac and H4K16ac, in good agreement with the aggregation plots (Fig. 4c). Little correlation was observed between H4K5ac and H4K8ac, consistent with their apparently distinct distributions by genome browser and aggregation plots.

### Methylation-specific antibodies

**Fig. 5:**
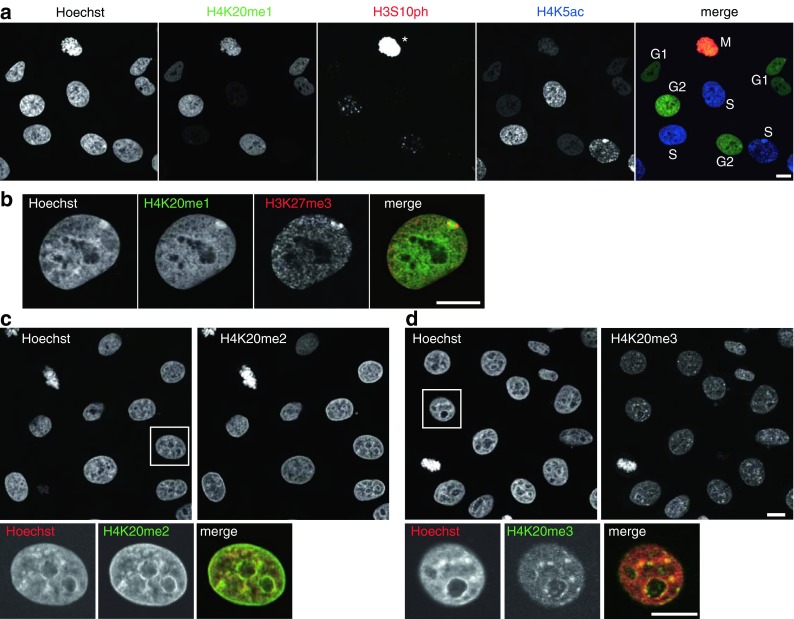
Localization of methylation-specific mAbs. hTERT-RPE1 (**a**, **b**) and HeLa (**c**, **d**) cells were fixed, immunolabeled with the indicated mAbs conjugated with Alexa Fluor 488, Cy3, or Cy5. DNA was counterstained with Hoechst 33342. Shown are single confocal sections. **a** hTERT-RPE1 cells were stained with H4K20me1-specific CMA421 (Alexa488), H3S10ph-specific CMA313 (Cy3), and H4K5ac-specific CMA405 (Cy5). The cell cycle points, judged from the staining patterns of H3S10ph and H4K5ac, are indicated. CMA421 is enriched in G2 cells (H3S10ph-positive; H4K5ac-negative) than cells in G1 (H3S10ph-negative; H4K5ac-negative) and S (H3S10-negative; H4K5ac-positive). Under the condition to detect H3S10ph signals in G2 cells, those on mitotic chromosomes are saturated (*asterisk*) due to massive phosphorylation during M phase. **b** hTERT-RPE1 cells were stained with H4K20me1-specific CMA421 (Alexa488) and H3K27me3-specific CMA323 (Cy3). CMA421 is concentrated on inactive X chromosome indicated by H3K27me3. **c** HeLa cells were stained with H4K20me2-specific CMA422 (Alexa488). CMA422 is distributed similarly to Hoechst 33342. **d** HeLa cells were stained with H4K20me3-specific CMA423 (Alexa488). CMA423 is concentrated on Hoechst-dense heterochromatin. *Bars*, 10 μm

Among the H4K20 methylation-specific mAbs, H4K20me1- and H4K20me3-specific mAbs (CMA421 and CMA423) reacted with their targets in the presence of K16ac, and H4K20me2-specific CMA422 hardly reacted with the peptide containing K16ac with K20me2 (Fig. 1). Immunoblotting showed these antibodies reacted specifically with the corresponding methylation mimic H4 proteins (Fig. 2). The specificity of each mAb to the target modification was also confirmed by their characteristic immunofluorescence patterns. hTERT-RPE1 cells stained with CMA421 showed heterogeneity in signal intensity (Fig. 5a), in good agreement with the previous report showing that H4K20me1 level increases during G2 to M phase (Rice et al. 2002; Pesavento et al. 2008). Indeed, interphase cells positive in H3S10 phosphorylation, which begins to appear in late S through G2, were often heavily stained with CMA421 (Fig. 5a). Thus, double staining cells with CMA405 (to detect S phase) and CMA421 (to detect G2 phase) was able to identify the cell cycle stage of individual cells (Fig. 5a). Furthermore, inactive X chromosomes were highlighted with CMA421 in hTERT-RPE1 cells (Fig. 5b), as shown previously. CMA422 and CMA423 exhibited DAPI-like and heterochromatin patterns, respectively, again consistent with previous observations (Fig. 5c, d).

## Conclusions

We showed that mouse mAbs directed against histone H4 and its modifications are highly specific to the targets and are applicable to immunoblotting, immunofluorescence, and ChIP-seq. While histone modification-specific antibodies are now widely available from different vendors, most of them are rabbit polyclonals and therefore associated with lot-to-lot variation of the specificity and affinity. Furthermore, a systematic study revealed that 20–25 % of histone modification-specific antibodies were disqualified for ChIP-seq application (Egelhofer et al. 2011). As the property of mAb remains constant over time, new H4-specific mAbs described here will be useful for future epigenetics and epigenome studies. In fact, some of these antibodies have already proven useful for immunofluorescence with mouse tissue sections (Eberhart et al 2012, 2013), histochemistry with human patient specimen (Yokoyama et al 2014; Shinchi et al 2015), and ChIP-seq using chicken DT40 cells (Hori et al 2014).

Among the mAbs described here, H4K5ac-specific mAb CMA405 has a unique specificity. It recognizes acetylated K5 with unacetylated K8. This combination is typically found in newly assembled H4, which is diacetylated at K5 and K12, and CMA405 indeed highlights structures that resemble DNA replication foci in S phase cells. Thus, CMA405 will be particularly useful for detecting newly assembled chromatin and also for highlighting cells in S phase by microscopy and flow cytometry.

## Abbreviations

HAT: Histone acetyltransferase
mAbs: Monoclonal antibodies
PBS: Phosphate-buffered saline
EdU: Ethynyl-deoxyuridine
TSSs: Transcription start sites
